# Treatment acceptability for disease‐modifying therapy for type 1 diabetes (T1D)—Views from parents of children with presymptomatic T1D

**DOI:** 10.1111/dom.16639

**Published:** 2025-07-23

**Authors:** Lauren M. Quinn, Olga Boiko, Josephine Elliott, Matthew Randell, Ian Litchfield, Felicity Boardman, Renuka P. Dias, Sheila M. Greenfield, Parth Narendran

**Affiliations:** ^1^ Institute of Immunology and Immunotherapy, University of Birmingham Brimingham UK; ^2^ Department of Applied Health Sciences University of Birmingham Brimingham UK; ^3^ Institute of Cancer and Genomic Sciences, University of Birmingham Birmingham UK; ^4^ Applied Health Directorate University of Warwick Warwick UK; ^5^ Department of Paediatric Endocrinology Birmingham Women and Children's Hospital Birmingham UK; ^6^ Department of Diabetes Queen Elizabeth Hospital Birmingham Birmingham UK

**Keywords:** attitudes, barriers, disease modifying therapy, perceptions, prevention, type 1 diabetes

## 1 BACKGROUND

The first disease‐modifying therapy for type 1 diabetes (T1D), teplizumab, became available in 2022 in the US.^1^ Teplizumab is currently undergoing review for licensing in the UK and delays symptomatic onset and need for insulin by approximately 2 years in those with stage 2 presymptomatic T1D,^2^ defined as multiple islet‐specific autoantibody seropositivity and dysglycaemia on oral glucose tolerance testing.^3^

Teplizumab is administered by daily infusion over 2 weeks, as a single course of treatment. Both hospital setting and home infusion can be accommodated.^1^ The most common adverse event is a mild cytokine release syndrome including myalgia, fever, and rash^2^ which resolves with prophylactic, symptomatic management.^1^ Approximately 70% develop transient lymphopenia within 30 days of treatment, which resolves without sequelae.^2^ Although appearing safe in the long term, with more than 6 years surveillance data demonstrating no increased risk of infection or malignancy in this cohort of patients,^4^ treatment comes at significant financial cost (both to healthcare systems and family) and requires absence from work and school.^5^ Critically, the acceptability of teplizumab treatment has not been formally assessed via qualitative interviews anywhere in the world and we wanted to explore this to outline important considerations for clinicians to support shared decision‐making should it be licensed in the UK. Here, we present the first qualitative data with parents/caregivers of stage 2 children, exploring views towards disease‐modifying treatment.

## 2 METHODS

The interviewed cohort comprised parents of stage 2 children who participated in the UK's EarLy Surveillance for Autoimmune diabetes (ELSA) study (www.elsadiabetes.nhs.uk, Ethics: 309252).^6^ Between 14th November 2022 and 2024, ELSA identified 22 children with stage 2 T1D from 22 families, all of whom were consecutively invited to interview by email, and two reminder emails were sent. Of these, 11 families (*n* = 12 parents) provided written informed consent and were interviewed (October 2023–October 2024). Nine interviews were completed within 3 months of stage 2 diagnosis, and two interviews within 6 months. Semi‐structured, video interviews were conducted by LQ, a clinical research fellow experienced in qualitative methods, who disseminated screening results and provided education on Early Stage T1D prior to the interview. We used a topic guide which is published in the protocol paper.^6^ At the start of the interview, the study aim was reiterated, that is, to understand parents' screening experience, including benefits, harms, and impact. Regarding teplizumab treatment, parents were reminded this can delay the need for insulin by 2 years on average, is administered as a daily intravenous infusion for 2 weeks, and is not currently licensed in the UK but is licensed in the US^6^. All interviews were undertaken prior to the availability of teplizumab via a pharmaceutical company managed access programme. Interviews were audio recorded and transcribed verbatim by a third party. Analysis commenced from the first interview, and data collection was discontinued following data saturation, that is, when no new insights were gained from the interview transcripts. Transcripts were iteratively coded, independently by two analysts (LQ and JE) and code books created in Microsoft Excel. Parents' open responses regarding their initial reaction to teplizumab were coded as acceptable (would currently consider accepting treatment for their child) or not acceptable (would not currently consider accepting treatment for their child). Inductive thematic analysis was performed according to Braun and Clark,^7^ initially with line‐by‐line open coding followed by axial coding to generate themes which fitted the dataset. Inductive thematic analysis was selected as this is a novel treatment area, and standardised frameworks for evaluating treatment acceptability are not yet established. For validity and transferability purposes, themes were refined in consultation with a multidisciplinary qualitative team, including a medical sociologist (SMG), medical ethicist (FB), senior qualitative research fellows (OB, IL) and clinicians (PN, RPD, JE, LQ). We identified three themes relating to screening acceptability, implications, and views towards disease‐modifying treatment. Here, we present an analysis of the third theme, to provide a detailed account of views, readiness, and possible treatment barriers (themes 1 and 2 will be reported elsewhere).^7^

## 3 RESULTS

Twelve parents/caregivers from 11 families participated in an interview where disease‐modifying treatments were discussed (one interview included two parents). Of these, 9/12 parents were White European, 10/12 had a family history of T1D (FHx), 9/12 were mothers and median parental age was 40 years (Table 1).^8^ Nine of the 12 parents had a child aged >8 years (*n* = 8 stage 2 children) and would be eligible for teplizumab treatment in the US.^1^

**TABLE 1:** Demographic characteristics.

Interview Number	Child eligible for teplizumab at time of interview	Age of child (years)	Country	Family history of T1D	Parent	Age of parent (years)	Occupation	Ethnicity
A1	Eligible	8	England	No	Mother	36	Health worker	White
A2		10	England	FDR	Mother	47	Administrator	White
A3		13	Scotland	FDR	Mother	38	Shop assistant	White
A4		8	Northern Ireland	FDR	Father	41	Civil Servant	White
A5		12	England	FDR	Mother	44	Finance officer	White
A6		10	England	FDR	Mother	43	Education Mental Health Practitioner	White
A7		9	England	SDR	Mother and Father	42 (Mother)	Coordinator/administrator (mother) and data analyst (father)	Other ethnic group—Arab; Any other ethnic group
A8		11	England	No	Mother	Not specified	Not specified	Not specified
A9	Ineligible	5	Wales	FDR	Mother	29	Senior electronics engineer	White
A10		6	England	FDR	Father	43	Structural engineer	Other ethnic group—Arab
A11		4	England	FDR	Mother	Not specified	Not specified	White

*Note*: Demographic characteristics of the interviewed parents. The cohort included 12 parents from 11 families.

Abbreviations: AXE, interview number; FDR, first degree relative; SDR, second degree relative; T1D, type 1 diabetes.

**FIGURE 1:**
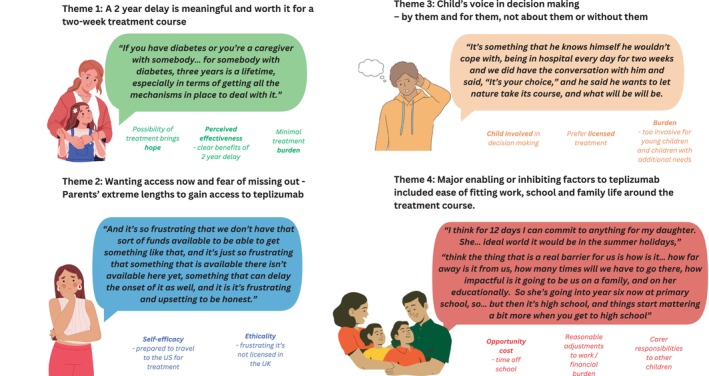
Views and considerations towards teplizumab. Acceptability for teplizumab treatment from parents of children with stage 2 type 1 diabetes. The four sub‐themes included: (1) value of a 2‐year delay to clinical diagnosis, (2) wanting access to treatment and fear of missing out, (3) child's role in parental decision making, and (4) ease of fitting work, school, and family life around the treatment course. Figure created using https://www.canva.com.

Overall, when asked about teplizumab, the majority of parents were interested in this treatment (*n* = 10/12, Table 2). All but one of the parents of an eligible child (8/9) would accept treatment if it were licensed in the UK. We identified four sub‐themes, including (1) value of delay to clinical diagnosis, (2) immediate access to treatment, (3) child's role in parental decision‐making, and (4) ease of fitting family life around the treatment course (Table 3, Figure 1).

**TABLE 2:** Parents' initial reaction when asked about teplizumab treatment.

Interview Number	Considered acceptable?	Initial reaction to teplizumab treatment
A1	Yes	“100% yeah definitely. I just… it frustrates me so much to be honest that it's not available here, because we're in that position. But yeah, 100%, anything that can delay the onset we're definitely open to looking at to be honest.”
A2	Yes	“that would be an amazing opportunity obviously for any parent to say okay we can delay this, because if you can delay it for even two/three years, how much in that two/three years are things going to come on where you can say okay now we've got something that could delay it for way longer.”
A3	Yes	“Yeah, I would consider that, and I think [daughter] would be quite keen to trial it. Again obviously we'd have to have a little read up about and get some information about any potential side effects and stuff, but I think yeah that's definitely something that we would be more likely to consider, a licensed one.”
A4	Yes	“It would be massive. Even I know if… even if I get away a day without having to look after my daughter, if my wife's looking after her, it's just it's life changing for them not having to worry about it, and I know I mentioned in the email but [son] is eight, going on eight and a half shortly, I think every year that we could delay it past now would be better for him,”
A5	Yes	“It would just make life a lot easier really. It's bad enough having to do it for myself, but having to do it for a child as well, because realistically although [daughter]'s nearly 13, she's not going to be able to be fully responsible for all of that, so a lot of it's going to come down to me. I don't want that for her, and I don't want it for us as a family, so if we can delay it… “
A6	Yes	“It would just make life a lot easier really. It's bad enough having to do it for myself, but having to do it for a child as well, because realistically although [daughter]'s nearly 13, she's not going to be able to be fully responsible for all of that, so a lot of it's going to come down to me. I don't want that for her, and I don't want it for us as a family, so if we can delay it…”
A7	Yes	“So I think we've spoken to… we're trying to find out a bit more about the TZIELD, and maybe doing that to delay the onset of type 1 diabetes. So we're in the process now of trying to set that up in the [hospital], where he will essentially have that for two weeks, over a two week period.”
A8	No	“[son] was completely adamant he doesn't want to do it. It's something that he knows himself he wouldn't cope with, being in hospital every day for two weeks and we did have the conversation with him and said, “It's your choice,” and he said he wants to let nature take its course, and what will be will be. “
A9	No	“I don't think she'd cope with it at all. She's been quite fortunate she's not been in hospital a lot, so I think she struggles with the whole environment. Like if it was an oral medication I think we'd probably have no bother about it at all, but I think because it's invasive I don't think she'd cope with it.”
A10	Yes	“Yes absolutely, yes, but I think from eight years old and older, not younger than that,”
A11	Yes	“I think that would be worth it for two years extra. I'd want to know more about it, but as a gut instinct I think it would be worth doing.”

*Note*: Supporting quotes for parental acceptability for teplizumab treatment.

Abbreviation: Tzield, teplizumab.

**TABLE 3:** Summary of sub‐themes regarding parents' views towards teplizumab.

	Sub‐theme	Explanation	Supporting quotes
1	Value of a 2–3 year delay to clinical type 1 diabetes	Two‐week treatment worth it for 2–3 years without clinical type 1 diabetes	“If you have diabetes or you're a caregiver with somebody… for somebody with diabetes, three years is a lifetime, especially in terms of getting all the mechanisms in place to deal with it. You're not rushing through trying to get pumps and your medication, and furniture, whatever. So three years is huge, it really is.” A4, Father of an 8 year old boy (FDR)
		Teplizumab offers a gateway to further potential treatments or future “cure”	“Absolutely, because as I said it could be a gateway to a further treatment, if we can keep her at stage two, and then they make another amazing development that… got to take any opportunity I think that is offered.” A2, Mother of a 10 year old girl (FDR)
		Appreciate time without having to manage day‐to‐day demands of type 1 diabetes	“It would just make life a lot easier really. It's bad enough having to do it for myself, but having to do it for a child as well, because realistically although [daughter]'s nearly 13, she's not going to be able to be fully responsible for all of that, so a lot of it's going to come down to me. I don't want that for her, and I don't want it for us as a family, so if we can delay it…” A5, Mother of a 12 year old girl (FDR) “I think for two weeks I think if that delayed the onset in my opinion it would be nothing for them two weeks, compared to what you're living with on a day to day life with diabetes. So for me the two weeks I think is not comparable really.” A1, Mother of an 8 year old girl (No family history of T1D) “I think anything that's going to delay the start of it is going to be powerful for her, to be able to have more time just being free from having a medical device stuck to your side, because she's going to go on a pump, they all go on pumps now, they all get sensors now, and hopefully by the time she gets they they'll be a bit smaller the sensors.” A6, Mother of a 10 year old girl (FDR)
		Delay could mean child is more mature and emotionally resilient at onset of clinical type 1 diabetes	“[son] is eight, going on eight and a half shortly, I think every year that we could delay it past now would be better for him, because he's becoming more mature and he's learning a lot more every day. It will give us the time too to educate him,” A4, Father of an 8 year old boy (FDR)
2	Wanting immediate access to treatment and fear of missing out	Frustrating it's not already available and family do not have the funds to pay for it	“It is, it's really frustrating to be honest. I think we spoke didn't we and said it was available in America for £200 000? And it's so frustrating that we don't have that sort of funds available to be able to get something like that, and it's just so frustrating that something that is available there isn't available here yet, something that can delay the onset of it as well, and it is it's frustrating and upsetting to be honest.” A1, Mother of an 8 year old girl (No family history of T1D)
		Fear of progression to Stage 3 and missing out on teplizumab	“But I think it would be frustrating for me if [daughter] was to then become insulin dependent and then not be able to have that treatment then, if that was approved months after she started the insulin, then I don't know how I would adjust to that to be honest.” A1, Mother of an 8 year old girl (No family history of T1D)
		Prepared to travel to US to get the treatment	“I was prepared to travel to the US if I had to, to get involved in these studies if it wasn't going to be in the UK and stuff like that. So there's I remember articulating it as I'm just prepared to do anything right now, just to help in whatever way is possible in terms of making this an easier transition for him.” A7, Mother and Father of a 9 year old boy (SDR)
		Prepared to travel long distances to UK nations/regions offering treatment	“even if he's not able to get it over here I'll travel to England to get it done. As long as we know when it's going to happen we can just take the time off work, we can take [son] out of school, whatever, and just get it done that way, it wouldn't be a problem.” A4, Father of an 8 year old boy (FDR)
		Preferred licensed treatment to research trials	“we'd have to have a little read up about and get some information about any potential side effects and stuff, but I think yeah that's definitely something that we would be more likely to consider, a licensed one.” A3, Mother of a 13 year old girl (FDR)
3	Child's role in decision‐making	Difficult making a decision on behalf of your child	“it's looking at the pros and cons and trying to weigh up that. There are things that worry me, so for example I know it can impact your white blood cell count, it will be for about a month he will be immunocompromised I guess during that time, and so it's… yeah it's worrying to know what is the right thing to do, and obviously because he's not old enough to make that decision you feel responsible to be making that, and just hoping.” A7, Mother and Father of a 9 year old boy (SDR)
		Child's ability to cope with the practical aspects of the treatment course	“It's something that he knows himself he wouldn't cope with, being in hospital every day for two weeks and we did have the conversation with him and said, “It's your choice,” and he said he wants to let nature take its course, and what will be will be.” A8, Mother of an 11 year old boy (No family history of T1D) “Daughter would not cope with daily infusions: ‘I don't think she'd cope with it at all. She's been quite fortunate, she's not been in hospital a lot, so I think she struggles with the whole environment. Like if it was an oral medication I think we'd probably have no bother about it at all, but I think because it's invasive, I don't think she's cope with it.” 091, Mother of a 5 year old girl (FDR)
		Needs to be acceptable to the child	“my husband's mum had pretty much every complication from diabetes possible, so it would be nice to delay it. But it would be [daughter]'s choice, it would be entirely up to her.” A9, Mother of a 5 year old girl (FDR)
4	Ease of fitting work, school/and family life around the treatment course	Main opportunity cost was disruption to schooling but parents offered reasonable adjustments	“think the thing that is a real barrier for us is how is it… how far away is it from us, how many times will we have to go there, how impactful is it going to be us on a family, and on her educationally. So she's going into year six now at primary school, so… but then it's high school, and things start mattering a bit more when you get to high school and she might be more wanting to stand out less and things like that.” A6, Mother of a 10 year old girl (FDR) “I think for 12 days I can commit to anything for my daughter. She… ideal world it would be in the summer holidays, and it won't be when we've just booked a holiday to go to Italy, it won't be then, it will be other times. But if you are saying if we get the opportunity to do this and it has to happen in May she has to go in May, she has to do it, and that's fine, we will do that, and school will do that, and we can do homework, and she can go in the afternoon. If the appointments are very early in the morning she can be at school by lunchtime if needs be. So yeah, or vice versa, if it's at the end of the day she can go to school in the morning and we can go at the end of the day… we would make it work for the opportunity to be able to do that.” A6, Mother of a 10 year old girl (FDR)
		Distance to treatment centre, flexibility in work schedule and financial burden associated with this, for example, travel, accommodation, time off work	“So logistically it's going to be a bit of a nightmare. My husband really can't take any time off, he has a very, very full on job. I do as well, but I'm term time only, I work in a school, so I'm hoping that I don't see how my employer could refuse for me to do it. But my plan is to essentially work from home whilst doing it around it as much as I can, and maybe if I need to make up the time later on. So it's going to be difficult, there's no getting round it, and for [daughter] again I'm hoping that for her as well they'll be able to give her some remote learning to do so that she's not completely missing two weeks of school and getting bored out of her mind.” A5, Mother of a 12 year old girl (FDR)
		Support from family members for siblings during the treatment period	“So… and then it was the fact that obviously we've got two other children, another one that's autistic at school and things like that, and my partner's working, it just wouldn't be… it's just not something we'd be able to do.” A8, Mother of an 11 year old boy (No family history of T1D)

*Note*: Summary of sub‐themes, supporting information and exemplar quotes from parents/guardians with a stage 2 child.

Abbreviations: AX, interview number; FDR, first degree relative (parent or sibling with type 1 diabetes); SDR, second degree relative (grandparent, aunt/uncle, cousin with type 1 diabetes); T1D, type 1 diabetes.

### 3.1 Value of delay to clinical diagnosis

I think for two weeks I think if that delayed the onset in my opinion it would be nothing for them two weeks, compared to what you're living with on a day to day life with diabetes. A1, Mother of an 8 year old girl with stage 2 T1D (no family history of T1D).

First, the 2‐week single treatment course was considered worthwhile for a 2‐year delay. Parents with lived experience of T1D imagined freedom from continuous glucose monitoring, carbohydrate counting and bolusing, alongside the benefits to the child's health across the life course from two additional diabetes‐free years. Any extra day without T1D was considered meaningful. Parents sought delay so their child would be more mature and emotionally resilient to better manage T1D. Teplizumab treatment also instilled a sense of hope—a 2‐year delay could provide time for additional treatments to become available to further delay, or even “*cure*”.

### 3.2 Immediate access to treatment

I'm just prepared to do anything right now, just to help in whatever way is possible in terms of making this an easier transition for him. A7, Mother and Father of a 9 year old boy with stage 2 T1D (has a second degree relative with T1D, SDR).

Second, parents wanted immediate access to teplizumab and many experienced fear of missing out. Awareness of the child's stage 2 status and increased risk of rapid progression to clinical T1D instilled a sense of urgency and responsibility to intervene. Opportunity to change the disease course and smooth the transition was viewed as a moral obligation. Parents worried their child may miss out and the licensing may come too late. They considered extreme lengths to access teplizumab, such as travelling to the US and using life savings to pay for treatment. Parents were prepared to take leave and travel significant distances to treatment centres.

### 3.3 Child's role in parental decision making

My husband's mum had pretty much every complication from diabetes possible, so it would be nice to delay it. But it would be [daughter]'s choice, it would be entirely up to her. A9, Mother of a 5 year old girl with stage 2 T1D (has a first degree relative with T1D, FDR).

Third, parents acknowledged the ethical challenges of making treatment decisions on behalf of their child. All parents wanted more detailed information on the side effects and risks to make an informed treatment decision. Yet, there was unequivocal preference for licensed treatment over trials, because the latter carried higher risk of unknowns, for example, side effects and uncertainty of benefit if placebo‐randomised. The child's voice in decision‐making was valued irrespective of age and parents were keen to make decisions with them, not for them. How the child would tolerate the treatment regimen was a major factor influencing acceptability. If the child was accepting of the practicalities of treatment, this increased parental acceptability. Conversely, two parents who declined teplizumab did so because the prospect of a 2‐week daily infusion was considered too distressing for the child due to needle phobia, fear of hospitals and/or additional needs, for example, autism spectrum disorder. These parents considered the treatment too intense for a 2‐year delay. However, if an oral equivalent was available, or the treatment could offer a lifelong cure, the cost/benefit calculation of undergoing treatment would be re‐evaluated.

### 3.4 Ease of fitting family life around the treatment course

Absolutely no problem if it is two weeks, it's not… and it's only an hour a day. I know I had talked to Doctor [Name] and Nurse [Name] about it, and I had said even if he's not able to get it over here I'll travel to England to get it done. As long as we know when it's going to happen we can just take the time off work, we can take [son] out of school, whatever, and just get it done that way, it wouldn't be a problem. A6, Father of an 8 year old boy with stage 2 T1D (FDR).

Fourth, treatment acceptability depended on the practicalities of fitting a 2‐week treatment into busy family life. Considerations included proximity and cost of travel to the treatment centre, and whether the family needed to arrange and pay for accommodation. The possibility of reasonable adjustments to the parents' work schedule was important, for example, home working or flexible hours. Parents wanted to minimise the impact on schooling, hoping the treatment could coincide with holidays and avoid examination periods, although parents recognised their child was agreeable to time off from school. Parents thought the school should provide homework so the child did not fall behind. Another non‐modifiable influencer was caring responsibilities for other children and whether they could rely on support from relatives during the treatment period. This emphasised the potential treatment burden of a disease‐modifying treatment for a presymptomatic disease and parents weighed this against the value of a possible 2 year delay to clinical diagnosis and insulin initiation.

## 4 DISCUSSION

Almost all interviewed parents considered teplizumab to be acceptable and were frustrated it was not already licensed in the UK. Teplizumab offered hope of postponing the demands of managing insulin‐requiring, symptomatic disease and reducing the total number of life years affected by T1D; all parents with an affected family member wanted access to treatment. The demands of the treatment course were considered minimal compared to these gains. Parents emphasised the child's role in decision‐making; teplizumab needed to be acceptable to both parent and child. The main opportunity cost was the potential negative impact on the child's schooling, but parents identified reasonable adjustments to minimise this. Parents sought more information about the risks and side effects of treatment, but the 2‐week treatment regimen was considered particularly burdensome for young children and those with additional needs.

In a US questionnaire study for adults and caregivers of stage 2 children who received teplizumab, 87% were grateful and >80% would recommend treatment, indicating similarly high treatment acceptability as observed in our interviewed cohort.^9^ Yet, an Italian questionnaire study with FDR parents reported lower acceptability, with only 52% of parents stating they would immediately consent to treatment if their child were eligible, but this was an unscreened (hypothetical) cohort.^10^ Therefore, it appears that treatment acceptability is higher once presymptomatic disease is established. Indeed, parents of children with stage 2 T1D called for urgent licensing whilst their child could still benefit. The value of delayed T1D onset was expressed in part by a willingness to pay; such sacrifices are observed in other paediatric chronic conditions such as juvenile idiopathic arthritis, where parents prioritised symptom improvement over financial cost, but nevertheless factored in school absences as an important opportunity cost.^11^ Similarly, caregivers' views towards disease‐modifying treatments, for example, for spinal muscular atrophy, instilled sentiments of hope and desperation to access treatment, driven by fear of progression and longing for a cure, observed more so at later disease stages.^12^, ^13^ Finally, parents thought it was essential to address treatment barriers, primarily to ensure equitable access and provide counselling to support informed decision‐making and an important life decision for the family, that is, both parent/caregiver and child.

The main limitation of this work is our cohort comprised of parents who completed a screening research programme (ELSA) and most had a T1D family history, were of White European ethnicity and lived in more affluent areas, thus the perspectives may not be representative of the UK general population. Although we include a small sample size, we reached data saturation within 11 interviews, which is previously shown to suffice when interviewing homogenous groups.^14^ Importantly, although only 50% of stage 2 families identified in the ELSA study completed the interview, their demographic characteristics did not differ from those who did not proceed to interview (Table 1, Table S1).

Despite these weaknesses, this study describes the priorities from the first cohort of UK parents with a child currently, or soon to become eligible for disease‐modifying therapies. These perspectives are timely and offer new insights into the end‐user acceptability of teplizumab, an essential consideration for both healthcare providers delivering therapy and professional stakeholders as treatment is becoming more widely available. Ongoing qualitative work from the ELSA study will aim to specifically explore attitudes from families without a T1D family history alongside children and young people's perspectives, which are critical to understand treatment acceptability at the population level. Meanwhile, screening programmes and the introduction of disease‐modifying treatments present significant challenges to healthcare systems, including cost of treatment, need for service restructuring, such as access to medical infusion suites, clinical support in case of side effects and psychological input to mitigate any negative psychosocial implications.^15^ Nevertheless, this study offers important insights into parental acceptability for disease‐modifying therapy for children at risk of T1D. We hope these insights will be valuable to policymakers as teplizumab, and potentially other therapies, become more widely available to people with T1D.

## AUTHOR CONTRIBUTIONS

L.M.Q., O.B., J.E., M.R., I.L., F.B., R.P.D., S.M.G. and P.N. researched data, contributed to discussion, wrote the first draft of the manuscript, and reviewed and edited the final manuscript. L.M.Q. is the guarantor of this work and, as such, had full access to all the data in the study and takes responsibility for the integrity of the data and the accuracy of the data analysis.

## FUNDING INFORMATION

The ELSA study is co‐funded by Diabetes UK and Breakthrough T1D (formerly the Juvenile Diabetes Research Foundation, JDRF), grant code: 20_0006315. The funders had no role in the study design; no role in data collection, analysis, and interpretation of data; no role in the writing of the report; and no role in the decision to submit the paper for publication.

## CONFLICT OF INTEREST STATEMENT

L.M.Q., O.B., J.E., M.R., I.L., F.B., and S.M.G. have no conflicts of interest to declare. R.P.D. has received honorarium from Sanofi (advisory board on Teplizumab). P.N. was a clinical expert for the National Institute of Clinical Excellence (NICE) on the development of teplizumab in January 2024 and has received an honorarium from Sanofi (advisory board on Teplizumab).

## PEER REVIEW

The peer review history for this article is available at https://www.webofscience.com/api/gateway/wos/peer-review/10.1111/dom.16639.

## Supporting information


**Data S1.** Supporting Information.

**Table S1.** Non‐interviewed parent demographics.

## Data Availability

The data analysed in this article cannot be shared openly because of necessary ethical restrictions to protect the participants’ identities. The data that support the findings of this study are, however, available from the authors upon reasonable request.
