# Supplementary material for: Treatment acceptability for disease‐modifying therapy for type 1 diabetes (T1D)—Views from parents of children with presymptomatic T1D
Source: Diabetes Obes Metab. 2025 Jul 23;27(10):6059–67. doi: 10.1111/dom.16639 (PMC12409228; doi:10.1111/dom.16639)
Supplement: Supplementary file 2 — Table S1. Non‐interviewed parent demographics. [file DOM-27-6059-s002.docx]

**Supplementary Table 1** – Non-interviewed parent demographics

| Non-interviewed participant number | Child eligible for teplizumab at time of interview | Age of child (years) | Country | Family history of T1D | Parent | Age of parent (years) | Occupation | Ethnicity of child |
| --- | --- | --- | --- | --- | --- | --- | --- | --- |
| 1 | no | 6 | Northern Ireland | Other Family member with T1D (half-sister) | Not collected* | | | White European |
| 2 | yes | 14 | England | FDR |  |  |  | White European |
| 3 | no | 5 | England | FDR |  |  |  | White European |
| 4 | yes | 11 | Scotland | FDR |  |  |  | White European |
| 5 | no | 5 | England | FDR |  |  |  | White European |
| 6 | no | 4 | Northern Ireland | FDR |  |  |  | White European |
| 7 | no | 6 | England | FDR |  |  |  | White European |
| 8 | yes | 10 | England | FDR |  |  |  | White European |
| 9 | yes | 8 | England | FDR |  |  |  | White European |
| 10 | yes | 10 | England | FDR |  |  |  | White European |
| 11 | yes | 11 | England | No |  |  |  | Black or Black British |

**Supplementary Table 1 legend** – Demographic characteristics of the parents with a stage 2 child who did not proceed to interview.

Key: T1D – type 1 diabetes, FDR – first degree relative with T1D, * demographic information was only collected from parents who consented to interview and for their personal information to be collected and stored.
